# An Electromyographic Analysis of Lateral Raise Variations and Frontal Raise in Competitive Bodybuilders

**DOI:** 10.3390/ijerph17176015

**Published:** 2020-08-19

**Authors:** Giuseppe Coratella, Gianpaolo Tornatore, Stefano Longo, Fabio Esposito, Emiliano Cè

**Affiliations:** 1 Department of Biomedical Sciences for Health, Università degli Studi di Milano, 20133 Milano, Italy; gianpaolo.tornatore@email.it (G.T.); stefano.longo@unimi.it (S.L.); fabio.esposito@unimi.it (F.E.); emiliano.ce@unimi.it (E.C.); 2 IRCSS Galeazzi Orthopaedic Institute, 20161 Milano, Italy

**Keywords:** muscle, EMG, deltoid, pectoralis major, upper trapezius, triceps brachii, resistance training, strength exercise

## Abstract

The present study examined the muscle activation in lateral raise with humerus rotated externally (LR-external), neutrally (LR-neutral), internally (LR-internal), with flexed elbow (LR-flexed) and frontal raise during both the concentric and eccentric phase. Ten competitive bodybuilders performed the exercises. Normalized surface electromyographic root mean square (sEMG RMS) was obtained from *anterior*, *medial*, and *posterior deltoid*, *pectoralis major*, *upper trapezius*, and *triceps brachii*. During the concentric phase, *anterior deltoid* and *posterior deltoid* showed greater sEMG RMS in frontal raise (effect size (ES)-range: 1.78/9.25)) and LR-internal (ES-range: 10.79/21.34), respectively, vs. all other exercises. *Medial deltoid* showed greater sEMG RMS in LR-neutral than LR-external (ES: 1.47 (95% confidence-interval—CI: 0.43/2.38)), frontal raise (ES: 10.28(95% CI: 6.67/13.01)), and LR-flexed (ES: 6.41(95% CI: 4.04/8.23)). *Pectoralis major* showed greater sEMG RMS in frontal raise vs. all other exercises (ES-range: 17.2/29.5), while *upper trapezius* (ES-range: 2.66/7.18) and *triceps brachii* (ES-range: 0.41/3.31) showed greater sEMG RMS in LR-internal vs. all other exercises. Similar recruitment patterns were found during the eccentric phase. When humerus rotates internally, greater activation of *posterior deltoid*, *triceps brachii*, and *upper trapezius* occurs. Humerus external rotation increases the activation of *anterior* and *medial deltoid.* Frontal raise mainly activates *anterior deltoid* and *pectoralis major*. LR variations and frontal raise activate specifically shoulders muscles and should be proposed accordingly.

## 1. Introduction

Resistance training is largely used in sports to increase muscle strength and promoting hypertrophic response [1] and in rehabilitation to reinforce muscles after injury [2]. Several exercises have been developed to target the agonist muscles through a series of mechanical stimuli; hence, a definition of the agonist muscle activation during each exercise may help to select the exercises appropriately [3,4]. Among the possible targets, the muscles surrounding the shoulders are involved in a series of multiplanar movements in many sports and daily life activities [5], so that several exercises are performed to reinforce these muscles. Particularly, *deltoid* muscle (which can be divided into *anterior*, *medial*, and *posterior deltoid*), *trapezius* (especially its upper portion), the clavicular head of *pectoralis major*, and *triceps brachii* are among the muscles targeted when performing shoulder exercises [5,6,7].

Lateral raise (LR) and frontal raise consist of arm abduction and flexion on the frontal or sagittal plane, respectively, and are largely used to stimulate the shoulders muscles [5,8,9,10]. LR can be performed with a range of variations, including an exercise where the humerus is rotated externally (LR-external), neutrally (LR-neutral), or internally (LR-internal) [5,10]. Comparing LR-external vs. LR-internal, a previous study reported that the latter elicited *medial* and *posterior deltoid* more than the former, while no data were observed for LR-neutral and *anterior deltoid* [10]. Another study also showed that the *upper trapezius* activation increased by rotating abducting the humerus on the sagittal vs. frontal plane [11]. Additionally, frontal raise is expected to mainly elicit *anterior deltoid* and the clavicular head of *pectoralis major*—two strong humerus flexors [5,11]. Interestingly, in practice, LR can be performed with a flexed elbow (LR-flexed). It is expected that LR-flexed might elicit less activation in *deltoid* muscles because of the shorter lever in frontal abduction; however, no study has compared these exercises so far. Lastly, *triceps brachii* is a strong stabilizer of the elbow during all LR variations and frontal raise. However, no study has examined its activation during these exercises. A comprehensive examination of the activation of the muscles surrounding the shoulders during LR variations and frontal raise may help to properly plan resistance training sessions to increase strength and possibly promote hypertrophy [12]. As such, each exercise could be selected depending on the muscle activation we want to focus on.

The previous studies investigating muscle activation in LR variations and frontal raise mainly recruited subjects with no-specified experience in strength training [6,8,10,11]. It has been shown that muscle activation for a given exercise is correlated with training experience [13] and possibly lead to different results. In contrast, competitive bodybuilders have a greater capacity to activate the agonist muscles while practicing strength exercises [3] and have shown already unique muscle pattern in bench press variations [14]. Consequently, investigating the differences in muscle activation across a range of similar exercises in subjects with greater training experience may elicit greater between-exercise differences in muscle recruitment comparing LR variations and frontal raise. Remarkably, resistance exercises usually consist of performing both the concentric and eccentric phases. Interestingly, performing repetitive eccentric phases incur into specific short-term [15,16] and long-term training-induced effects [1,17,18] compared to concentric-based training. Hence, examining the muscle activation in LR variations and frontal raise during both the concentric and the eccentric phase could further differentiate the training stimuli through an accentuation of the concentric or eccentric phase. To date, only one study investigated the muscle recruitment during the eccentric phase of isokinetic shoulder abduction, highlighting that overall muscle activation was influenced by velocity and torque exerted, but no comparison was done with the concentric phase [9]. Therefore, the present study aimed to examine the muscle activation of *deltoid*, *upper trapezius*, the clavicular head of *pectoralis major*, and *triceps brachii* in a range of LR variations (LR-external, LR-neutral, LR-internal, LR-flexed) and frontal raise during both the concentric and eccentric phase in competitive bodybuilders.

## 2. Material and Methods

### 2.1. Study Design

The present investigation was designed as a cross-over, repeated-measures, within-subject study. The participants were involved in three different sessions. In the first session, the participants were familiarized with the technique of each selected exercise. In the second session, the load for each exercise was determined. In the third session, the muscles’ maximum activation was measured; thereafter, after a minimum of 30 min of passive recovery, the participants performed each exercise in random order. Each session was separated by at least three days, and the participants were instructed to avoid any further form of resistance training for the entire duration of the investigation.

### 2.2. Participants

The present procedures were advertised by the operators during some regional and national competitions, and, to be included in the study, the participants had to compete in regional competitions for a minimum of at least 5 years. Additionally, they had to be clinically healthy, without any reported history of upper-limb and trunk muscle injury and neurological or cardiovascular disease in the previous 12 months. To avoid possible confounding factors, the participants competed in the same weight category (Men’s Classic Bodybuilding <80 kg, <1.70 m), according to the International Federation of Body Building Pro-League. The use of drugs or steroids was continuously monitored by a dedicated authority under its regulations, albeit we could have not controlled for it. Thereafter, 10 male competitive bodybuilders (age 29.8 ± 3.0 yrs; body mass 77.9 ± 1.0 kg; stature 1.68 ± 0.01 m; training seniority 10.6 ± 1.8 yrs) were recruited for the present investigation. The participants were asked to abstain from alcohol, caffeine, or similar beverages in the 24 h preceding the test. After a full explanation of the aims of the study and the experimental procedures, the participants signed written informed consent. They were also free to withdraw at any time. The procedures were approved by the Ethical Committee of the Università degli Studi di Milano (CE 27/17) and performed in accordance with the Declaration of Helsinki (1975) for studies involving human subjects.

### 2.3. Maximus Voluntary Isometric Activation

The maximal voluntary isometric activation of *anterior deltoid*, *medial deltoid*, *posterior deltoid*, the clavicular head of *pectoralis major*, *upper trapezius*, and lateral head of *triceps brachii* was measured in random order. The electrodes placement followed the SENIAM (surface electromyography for the non-invasive assessment of muscles) recommendations [19]. The surface electromyographic (sEMG) electrode for the *anterior deltoid* was placed over the mid-belly of the muscle approximately 4 cm below the clavicle [19]. The *medial deltoid* had 2 electrodes placed on the lateral aspect of the deltoid, 3 cm below the acromion process [19]. For *posterior deltoid*, the electrodes were placed in the area about two fingerbreadths behind the angle of the acromion [19]. The sEMG electrodes for the clavicular head of the *pectoralis major* were placed on the midclavicular line, midway between the acromioclavicular joint of the shoulder for the clavicular head [19]. For *upper trapezius*, the electrodes were placed at 50% on the line from the acromion to the spine on vertebra C7 [19]. For the lateral head of *triceps brachii*, the electrodes were placed at 50% on the line between the posterior crista of the acromion and the olecranon at two finger widths lateral to the line [19]. The electrodes were placed on the dominant limb [14]. To check for appropriate electrodes placement previous procedures were followed [14]. For example, if the electrode shifted over the innervation zone during part of the movement, the EMG amplitude was underestimated. Therefore, to check for any consequence due to a possible shift of the surface electrode over the innervation zone, a Fast-Fourier Transform approach was used, as suggested in a previous investigation [20]. Briefly, the electrode placement on each muscle was checked during the warm-up phase of each exercise, analyzing the power spectrum profile of the sEMG signal recorded at the starting-, middle-, and end-point of each exercise in all muscles. The correct electrode placement results in a typical belly-shaped power spectrum profile of the EMG signal, while noise, motion artifacts, power lines, and electrodes placed on the innervation zone or myotendinous junction generate a different power spectrum profile [20]. If the power spectrum did not match with the typical belly-shaped power spectrum profile in any of the temporal points, the electrodes were repositioned, and the procedures repeated so to have a clear EMG signal from all the muscles throughout the movement. The same experienced operator placed the electrodes and checked the power-spectrum profile. This approach was shown to provide very high reliability in sEMG data [14].

To determine the maximal voluntary activation of all selected muscles, the participants were asked to exert their maximal force against unmovable or manual resistance. For the *anterior deltoid*, the participants were instructed to flex the elbow to 90° so that the hand was pointed upwards. Then, they were asked to make a closed fist with the hand of the flexed arm and to provide maximal force to produce shoulder flexion against manual resistance [14]. For the *medial deltoid*, the participants were instructed to flex the elbow to 90° and were asked to maximally abduct the flexed arm against manual resistance [14]. For the *posterior deltoid*, the participants were asked to abduct the shoulder in a slight extension against manual resistance, with the humerus in slight internal rotation [19]. For *upper trapezius*, the participants were instructed to elevate the acromial end of the clavicula and scapula against unmovable resistance pushing downward [19]. For the clavicular head of the *pectoralis major*, the participants were instructed to horizontally abduct the arm with the shoulder and elbow flexed at 90°. Then, they provided maximal force while attempting to horizontally adduct the arm against unmovable resistance [14]. For the lateral head of *triceps brachii*, the participants were instructed to extend the elbow against unmovable resistance toward the elbow flexion [19]. Each attempt lasted 5 s, and three attempts were completed for each movement interspersed by 3 min of passive recovery [14]. The operators provided strong standardized verbal encouragement.

The electrodes were equipped with a probe (probe mass: 8.5 g, BTS Inc., Milano, Italy) that permitted the detection and the transfer of the sEMG signal by wireless modality. sEMG signal was acquired at 1000 Hz, amplified (gain: 2000, impedance and the common rejection mode ratio of the equipment are >1015 Ω//0.2 pF and 60/10 Hz 92 dB, respectively), and driven to a wireless electromyographic system (FREEEMG 300, BTS Inc., Milano, Italy) that digitized (1000 Hz) and filtered (filter type: IV-order Butterworth filter; bandwidth: 10–500 Hz) the raw sEMG signals.

### 2.4. Exercise Protocols

Given the nature of each exercise and to avoid any possible involvement of muscles not directly involved in the movement during the exercise execution (e.g., lumbar muscles), each exercise was performed seated with a load that allowed eight repetitions (i.e., 8-RM). The 8-RM was determined, incrementing the load in accordance with previous procedures [21,22]. Once the 8-RM was determined, the participants performed six repetitions for each exercise to avoid the effects of fatigue on the sEMG signal [14]. Each exercise was performed with the pacing of 2 s for the concentric and 2 s for the eccentric phase and 0.5 s for the isometric phase at the beginning and at the end of each movement. A metronome was used to pace the intended duty cycle, and a camera was used to provide feedback about each exercise technique. A standardized warm-up consisting of 10 clockwise + 10 counterclockwise arms circling preceded the data collection.

**Figure 1:**
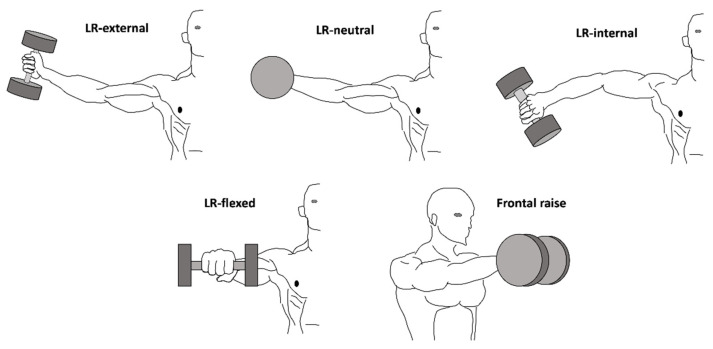
A schematic representation of the technique of each exercise is shown.

All exercises were performed avoiding any movement in elbow and wrist flexion/extension or wrist pronation/supination. For LR-external, the participants were required to abduct laterally the humerus up to 90°, with humerus externally rotated (i.e., thumbs up). For LR-neutral, the participants were required to abduct laterally the humerus up to 90°, with the humerus neutrally rotated (i.e., thumbs forward). For LR-internal, the participants were required to abduct laterally the humerus up to 90°, with the humerus internally rotated (i.e., thumbs down). For frontal raise, the participants were required to frontally flex the humerus up to 90°, with humerus neutrally rotated (i.e., thumb-to-thumb). For all these exercises, the elbow was almost fully extended, and the wrist in line with the forearm [11]. For LR-flexed, the participants were required to abduct laterally the humerus up to 90°, with the elbow flexed at 90° and the humerus neutrally rotated (i.e., thumb-to-thumb). A schematic representation of the technique of each exercise is depicted in Figure 1.

### 2.5. Data Analysis

The sEMG signals from both the maximal voluntary isometric activation and separately from the concentric and eccentric phases of each exercise were analyzed in time-domain, using a 25-ms mobile window for the computation of the root mean square (RMS). For the maximal voluntary isometric activation, the average of the RMS corresponding to the central 2 s was considered. During each exercise, the RMS was calculated and averaged separately over the 2 s of the concentric and eccentric phase. Out of the six repetitions, we excluded the first and the last to avoid any possible confounding factor related to the beginning and the end of each set and to further exclude any symptom of fatigue. Thereafter, the sEMG RMS of each muscle during each exercise was normalized for its respective maximal voluntary isometric activation [13,14,23,24] and inserted into the data analysis.

### 2.6. Statistical Analysis

The statistical analysis was performed using statistical software (SPSS 22.0, IBM, Armonk, NY, USA). The normality of data was checked using the Shapiro–Wilk’s test, and all distributions were normal. Descriptive statistics are reported as mean (SD). The intrasession sEMG RMS reliability was calculated using an intra-class coefficient (ICC), and the standard error of the measurement expressed as percentage variability (SEM%) for each muscle maximum voluntary isometric activation and for the concentric and eccentric phase of each exercise comparing the second and the fifth repetition. The ICC was interpreted as follows: ≥0.90: very high; 0.89–0.70: high; 0.69–0.50: moderate. The differences in the normalized sEMG RMS were separately calculated for each muscle between the exercise (LR-external, LR-neutral, LR-internal, frontal raise, and LR-flexed) and phase (2 levels: concentric and eccentric) using a two-way ANOVA. Multiple comparisons were adjusted using the Bonferroni’s correction. Significance was set at *p* < 0.05. Eta squared (η^2^) and partial η^2^ were calculated to estimate the degree of variance of the dependent factor due to the independent factors and interpreted as follows: <0.059: small; 0.06 to 0.12: medium; >0.13: large [25]. The Cohen’s *d* effect size (ES) with 95% confidence interval (CI) was reported for significant pairwise comparisons and interpreted according to the Hopkins’ recommendations: 0–0.19: trivial; 0.20–0.59: small; 0.60–1.19: moderate; 1.20–1.99: large; ≥2.00: very large [26].

## 3. Results

The intrasession reliability of the sEMG RMS signal for muscle maximum voluntary isometric activation and for the concentric and eccentric phase of each exercise is provided in Table 1. ICC and SEM% during the maximum activation ranged from 0.878 to 0.927 and 2.7% to 6.6%, respectively. During each exercise, ICC and SEM% were comprised between 0.795 and 0.904 and between 5.4% and 8.7% during the concentric phase, whereas spanned from 0.798 to 0.903 and 5.2% to 8.5% during the eccentric phase.

**Figure 2:**
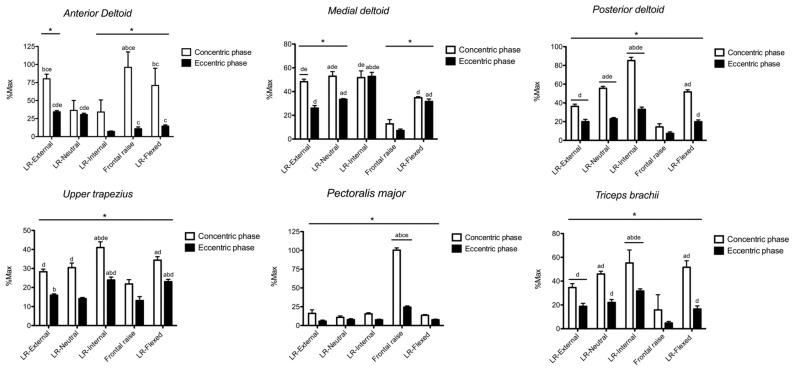
The normalized to maximum voluntary activation (%Max) surface electromyography root mean square is shown for each muscle during both the concentric and eccentric phase for each exercise. LR: lateral raise *: concentric > eccentric phase (*p* < 0.05), a: greater than LR-external rotation (*p* < 0.05), b: greater than LR-neutral rotation (*p* < 0.05), c: greater than LR-internal rotation (*p* < 0.05), d: greater than frontal raise (*p* < 0.05), e: greater than LR-flexed elbow (*p* < 0.05).

The sEMG RMS amplitude for *anterior deltoid* is shown in Figure 2. Exercise × phase interaction (*p* < 0.001, F = 493.3, η^2^ = 0.998) was found. With the exception of the LR-neutral, greater (*p* < 0.05, F = 388.6, partial η^2^ = 0.982) sEMG RMS was found during the concentric vs. eccentric phase in all other exercises. During the concentric phase, sEMG RMS was greater in frontal raise compared with LR-external (ES: 1.78, from 0.63 to 2.73), LR-neutral (ES: 5.30, from 3.27 to 6.87), LR-internal (ES: 6.72, from 4.26 to 6.82), and LR-flexed (ES: 1.95, from 0.81 to 2.91). LR-external showed greater sEMG RMS than LR-neutral (ES: 4.68, from 3.20 to 5.91), LR-internal (ES: 6.84, from 4.82 to 8.48), and LR-flexed (ES: 0.82, from 0.05 to 1.84). LR-flexed showed greater EMG RMS than LR-neutral (ES: 4.68, from 2.84 to 6.13) and LR-internal (ES: 6.84, from 4.34 to 8.76). During the eccentric phase, sEMG RMS in LR-external and LR-neutral was greater than LR-internal (ES: 13.04, from 8.72 to 16.46 and 10.66, from 6.92 to 13.49, respectively), frontal raise (ES: 5.94, from 3.72 to 7.95 and 4.31, from 2.68 to 5.68, respectively), and LR-flexed (ES: 6.19, from 3.89 to 7.97 and 3.88, from 2.27 to 5.17, respectively). Frontal raise and LR-flexed had greater sEMG RMS than LR-internal (ES: 2.55, from 1.28 to 3.60 and 5.70, from 3.55 to 7.37, respectively). No further difference was observed.

The sEMG RMS amplitude for *medial deltoid* is shown in Figure 2. Exercise × phase interaction (*p* = 0.002, F = 35.7, η^2^ = 0.973) was found. With the exception of LR-internal, greater (*p* < 0.05, F = 380.8, partial η^2^ = 0.982) sEMG RMS was found during the concentric vs. eccentric phase. During the concentric phase, sEMG RMS was greater in LR-neutral than LR-external (ES: 1.47, from 0.43 to 2.38), frontal raise (ES: 10.28, from 6.67 to 13.01), and LR-flexed (ES: 6.41, from 4.04 to 8.23). Both LR-external and LR-internal had greater sEMG RMS than frontal raise (ES: 11.20, from 7.29 to 14.17 and ES: 8.00, from 5.13 to 10.19, respectively) and LR-flexed (ES: 8.10, from 5.20 to 10.32 and ES: 4.17, from 2.48 to 5.51, respectively). LR-flexed showed greater sEMG RMS than frontal raise (ES: 8.10, from 5.20 to 11.00). During the eccentric phase, LR-internal showed greater sEMG RMS than LR-external (ES: 9.32, from 6.03 to 11.83), LR-neutral (ES: 7.85, from 5.03 to 10.01), frontal raise (ES: 17.69, from 11.62 to 22.26), and LR-flexed (ES: 7.39, from 4.71 to 9.44). Both LR-neutral and LR-flexed had greater sEMG RMS than LR-external (ES: 4.58, from 2.77 to 6.00 and ES: 2.53, from 1.27 to 2.58, respectively) and frontal raise (ES: 15.62, from 6.88 to 22.19 and ES: 13.60, from 8.89 to 17.15, respectively). LR-external showed greater sEMG RMS than frontal raise (ES: 10.50, from 6.82 to 13.30). No further difference was observed.

The sEMG RMS amplitude for *posterior deltoid* is shown in Figure 2. Exercise × phase interaction (*p* < 0.001, F = 234.4, η^2^ = 0.996) was found. Whatever the exercise, greater (*p* < 0.05, F = 151.9, partial η^2^ = 0.995) sEMG RMS was found during the concentric vs. eccentric phase. During both the concentric and eccentric phase, LR-internal showed greater sEMG RMS compared with LR-external (ES: 16.48, from 10.72 to 20.86 and ES: 5.36, from 3.31 to 6.95, respectively), LR-neutral (ES: 10.34, from 6.71 to 13.09 and ES: 5.13, from 3.16 to 6.67, respectively), frontal raise (ES: 20.72, from 13.63 to 26.05 and ES: 12.28, from 6.01 to 18.51, respectively), and LR-flexed (ES: 11.41, from 7.43 to 14.42 and ES: 6.04, from 3.79 to 7.78, respectively). During both concentric and eccentric phase, LR-neutral showed greater sEMG RMS than LR-external (ES: 8.69, from 5.60 to 11.05 and ES: 1.51, from 0.46 to 2.43 respectively), frontal raise (ES: 14.74, from 9.66 to 18.58 and ES: 10.21, from 6.62 to 14.93, respectively), and LR-flexed (ES: 1.80, from 0.69 to 2.75 and ES: 1.89, from 0.77 to 2.85, respectively). During the concentric phase, LR-flexed showed greater sEMG RMS than LR-external (ES: 6.65, from 4.21 to 8.82) and frontal raise (ES: 12.99, from 8.49 to 16.39), and LR-external had greater sEMG RMS than frontal raise (ES: 7.55, from 4.82 to 9.63). During the eccentric phase, both LR-external and LR-flexed showed greater sEMG RMS than frontal raise (ES: 5.99, from 3.75 to 7.92 and ES: 6.89, from 4.37 to 8.82, respectively). No further difference was observed.

The sEMG RMS amplitude for *upper trapezius* is shown in Figure 2. Exercise × phase interaction (*p* = 0.002, F = 37.3, η^2^ = 0.974) was found. Whatever the exercise, greater (*p* < 0.05, F = 165.8, partial η^2^ = 0.996) sEMG RMS was found during the concentric vs. eccentric phase. During the concentric phase, sEMG RMS was greater in LR-internal compared with LR-external (ES: 5.43, from 3.37 to 7.04), LR-neutral (ES: 3.86, from 2.26 to 5.14), frontal raise (ES: 7.18, from 4.97 to 9.18), and LR-flexed (ES: 2.66, from 1.36 to 3.72). LR-flexed had greater sEMG RMS than LR-external (ES: 3.72, from 2.15 to 4.97) and frontal raise (ES: 6.03, from 3.78 to 7.77). Both LR-external and LR-neutral had greater sEMG RMS than frontal raise (ES: 3.44, from 1.95 to 4.63 and ES: 3.65, from 2.10 to 4.89, respectively). During the eccentric phase, both LR-internal and LR-flexed had greater sEMG RMS compared with LR-external (ES: 6.50, from 4.11 to 8.34 and ES: 7.11, from 4.53 to 9.10, respectively), LR-neutral (ES: 8.15, from 5.23 to 10.38 and ES: 9.26, from 5.98 to 11.75, respectively), and frontal raise (ES: 5.84, from 3.65 to 7.54 and ES: 5.83, from 3.65 to 7.53, respectively). No further difference was observed.

The sEMG RMS amplitude for *pectoralis major* is shown in Figure 2. Exercise × phase interaction (*p* < 0.001, F = 111.4, η^2^ = 0.999) was found. Whatever the exercise, greater (*p* < 0.05, F = 115.2, partial η^2^ = 0.996) sEMG RMS was found during the concentric vs. eccentric phase. During the concentric phase, sEMG RMS was very largely greater in frontal raise compared with LR-external (ES: 21.31, from 14.03 to 26.70), LR-neutral (ES: 29.90, from 19.72 to 37.55), LR-internal (ES: 30.39, from 20.04 to 38.16), and LR-flexed (ES: 34.21, from 22.57 to 42.96). During the eccentric phase, sEMG RMS of clavicular was very largely greater in frontal raise compared with LR-external (ES: 11.61, from 7.57 to 14.68), LR-neutral (ES: 11.01, from 7.16 to 13.92), LR-internal (ES: 12.46, from 8.13 to 15.74), and LR-flexed (ES: 12.36, from 8.07 to 15.61). No further difference was observed.

The sEMG RMS amplitude for *triceps brachii* is shown in Figure 2. Exercise × phase interaction was found (*p* = 0.003, F = 28.2, η^2^ = 0.966). Whatever the exercise, greater (*p* < 0.05, F = 229.0, partial η^2^ = 0.970) sEMG RMS was found during the concentric vs. eccentric phase. sEMG RMS was greater in LR-internal during the concentric and eccentric phase compared with LR-external (ES: 2.54, from 1.28 to 3.59 and ES: 5.81, from 3.63 to 7.50, respectively), LR-neutral (ES: 1.17, from 0.17 to 2.06 and ES: 4.45, from 2.67 to 5.84, respectively), frontal raise (ES: 3.31, from 1.86 to 4.49 and ES: 17.07, from 11.21 to 21.49, respectively), and LR-flexed (ES: 0.91, from 0.09 to 1.78 and ES: 6.73, from 4.26 to 8.62, respectively). During the concentric phase, LR-external and LR-flexed showed greater sEMG RMS than LR-external (ES: 3.98, from 2.66 to 5.08 and ES: 3.78, from 2.50 to 4.85, respectively) and frontal raise (ES: 3.29, from 2.12 to 4.29 and ES: 3.66, from 2.41 to 4.71, respectively). LR-external had greater sEMG RMS than frontal raise (ES: 2.02, from 1.09 to 2.84). During the eccentric phase, sEMG RMS was greater in LR-external (ES: 6.93, from 4.40 to 8.87), LR-neutral (ES: 8.81, from 5.86 to 11.20), and LR-flexed (ES: 5.85, from 3.66 to 7.55) vs. frontal raise. No further difference was observed.

## 4. Discussion

The present study investigated the sEMG RMS in several agonist muscles in a range of shoulder raise exercises during the concentric and eccentric phase of each exercise. During the concentric phase, sEMG RMS in *anterior deltoid* was greater during frontal raise and LR-external. *Medial deltoid* was more active during the lateral raises, particularly when the humerus had a neutral or internal rotation. *Posterior deltoid* had greater sEMG RMS in LR-internal compared with all other exercises. *Upper trapezius* had greater sEMG RMS during LR-internal compared with all other exercises. The clavicular head of the *pectoralis major* was more active during frontal raise compared with all other exercises. Lastly, the lateral head of *triceps brachii* was more active in LR-internal compared with all other exercises. Although this muscle activity pattern was overall similar during the eccentric phase, *anterior deltoid* had similar activation in LR-external and LR-neutral and *medial deltoid* in LR-internal compared with all other exercises. The present findings showed unique muscle activation for each LR variations and frontal raise. Practitioners should thus select these exercises depending on the shoulders muscles that need to be activated at each occasion.

LR-external, LR-neutral, and LR-internal are performed abducting the humerus, which is rotated, respectively, externally, neutrally, or internally for the entire range of motion. For LR-external, the present results showed that the deltoids sEMG RMS was ~80% in *anterior*, > ~48% in *medial*, > ~36% in the *posterior* head. For LR-neutral, sEMG RMS was similar in *medial* and *posterior* (~55% and ~52%), but lower in *anterior deltoid* (~36%). For LR-internal, sEMG RMS was ~85% in *posterior*, ~52% in *medial*, and ~34% in *anterior* deltoid, in line with previous results [10]. Although *anterior*, *medial*, and *posterior deltoids* act synergistically as humerus abductors on the frontal plane, *anterior deltoid* is an internal, while *posterior deltoid* is an external rotator of the humerus [5]. The internal or external rotation of the humerus generates an increment in moment arm for *posterior* and *anterior* deltoid, respectively, thus increasing their activity [5]. Additionally, bi-articular muscles show greater sEMG RMS at long compared with short muscle length [27,28], possibly because of changes in moment arm and muscle architecture [29], modifications in both spinal and supraspinal mechanisms [30], or also changes in the characteristics of motoneurons pool when a muscle is lengthened [28]. Interestingly, although a computational study found that the humerus rotation could have led to negligible differences, here, the present competitive bodybuilders might have been able to increase the muscle activation for each exercise [3]. Therefore, the present results seemed to confirm the data reported in the literature. It should be noted that, during the eccentric phase, *anterior deltoid* showed similar sEMG RMS in LR-external vs. LR-neutral, possibly because of the greater control needed to decelerate the humerus, as reported previously [9]. Similarly, during the eccentric phase, the greatest *medial deltoid* activity was found in LR-internal, which possibly needs greater control [8]. The *triceps brachii* activation was overall greater in LR-internal > LR-neutral > LR-external. The greater the internal rotation of the humerus, the greater the weight of the forearm + wrist + dumbbell system becomes against the gravity. Indeed, the internal rotation of the humerus lets the elbow’s mobility be free against the gravity, whereas the external rotation puts the elbow’s extension in favor of the gravity, fixing the elbow joint. Therefore, because all exercises were performed at a fixed elbow angle, *triceps brachii* increased its elbow-stabilizer role when rotating internally the humerus. The internal rotation of the humerus also increased the role of *upper trapezius* in LR-internal > LR-neutral/LR-external. Indeed, when rotating internally the humerus, the scapulae are elevated and rotated upward [11], thus augmenting the *upper trapezius* activity. Lastly, the clavicular head of the *pectoralis major* showed similar low activity in all LR variations, indicating that it plays a marginal role when the humerus is abducted laterally.

LR-flexed, compared to the other LR variations, was performed with the flexed elbow at 90°, and the humerus rotated neutrally. However: (i) the length of the lever on the frontal plane was shorter than the other LR-variations, and (ii) the forearm was placed forward on the sagittal plane. Firstly, considering the point (i), the sEMG RMS of *medial deltoid* was hence lower compared to the other LR variations, where the arm was lifted almost straight on the frontal plane, increasing the length of the lever. Secondly, considering the point (ii), the forearm placed forward on the sagittal plan increased the activity of *anterior deltoid* as frontal humerus flexors. Indeed, greater sEMG RMS compared to LR-neutral and LR-internal was found here. Moreover, because of the forward weight that leads the humerus to rotate internally, the *posterior deltoid* had to counteract as an external rotator, thus stabilizing the rotation of the humerus [29]. Therefore, the sEMG RMS was similar to LR-neutral and greater than LR-external. Such an internal rotation of the humerus also led to elevate the external rotation of the scapulae [5], so that *upper trapezius* was more active during both the concentric and eccentric phase compared to LR-external and LR-neutral. It should be also noted that the flexed elbow increased the muscle length at which *triceps brachii* had to contract to stabilize the elbow joint [27]. This might have led to similar and greater sEMG RMS in LR-flexed compared with LR-neutral and LR-external, respectively, in line with previous results [27]. Lastly, the clavicular head of the *pectoralis major* seemed to play a marginal role in LR-flexed, as pointed out by the low level of activation.

Frontal raise was performed on the sagittal plane, lifting up the upper limb in neutral rotation by a frontal flexion, with a straight elbow. As a whole, frontal raise appears to mainly require the activation of the *anterior deltoid* and the clavicular head of the *pectoralis major*. Indeed, both muscles showed the greatest activity compared with all the remaining exercises. In line, when the humerus moved towards a flexion across the sagittal plane, the sEMG RMS in *anterior deltoid* and the clavicular head of the *pectoralis major* was reported to increase [14]. In contrast, given the low activation level recorded here, both *medial* and *posterior deltoids* appeared to play a negligible role in frontal raise, in line with previous results [9]. *Upper trapezius* showed relatively low activity, possibly because, when flexing the humerus, the scapulae tend to rotate internally and to be depressed [11]. Interestingly, albeit the least active compared with all exercises during the concentric phase, the sEMG RMS in *upper trapezius* was similar to LR-external and LR-neutral during the eccentric phase. This might depend on the need to stabilize the scapula when the upper limb was accelerating downward, as previously reported [9]. Lastly, *triceps brachii* showed poor activation, possibly due to the neutral humerus rotation that *per se* stabilized the elbow joint on the craniocaudal axis.

Some limitations accompany the present procedures. Firstly, the data shown are specific for the population involved and the technical requirements provided to perform each exercise. Nonetheless, although it is acknowledged that different populations may result in different findings, the present population made us confident about the goodness of the technique and muscle recruitment. Similarly, even though greater load could result in different outcomes, this may also reduce the quality of the technique execution for each exercise, and we are confident that here the load was suitable for the population. Moreover, although a dedicated authority continuously monitored the use of steroids, we could not have controlled for, and we do not know how the possible use of steroids would affect muscle activation. Lastly, some authors have divided *deltoid* muscle into seven different parts [31]. However, sEMG was not able to distinguish each of them, so we made the traditional 3-part subdivision.

## 5. Conclusions

In conclusion, different LR variations and frontal raise had unique muscle recruitment when performed by competitive bodybuilders. Concerning LR, *anterior deltoid* increased its activity when the humerus was rotated externally, while *posterior deltoid* when the humerus was rotated internally. *Medial deltoid* was more active during LR with straight vs. flexed elbow. Frontal raise strongly recruited *anterior deltoid* and the clavicular head of *pectoralis major*. *Triceps brachii* was involved in stabilizing the elbow, and greater activity occurred when the humerus was rotated internally or the elbow was flexed. Lastly, *upper trapezius* showed greater activity when humerus tended to be rotated internally.

## Figures and Tables

**Table 1:** Intrasession reliability (ICC and SEM%) of surface electromyography root mean square value calculated during the maximum voluntary isometric test and during the concentric and eccentric phase of each exercise.

	Maximum Voluntary Isometric Activation				Muscle Activation During Exercises (2nd vs. 5th Rep)			
					Concentric Phase		Eccentric Phase	
Muscle	Trial Min ($\bar{m}$ ± SD)	Trial Max ($\bar{m}$ ± SD)	ICC	SEM%	ICC (Min–Max)	SEM% (Min–Max)	ICC (Min–Max)	SEM% (Min–Max)
*Anterior deltoid*	0.885 ± 0.148	0.891± 0.103	0.927	3.8	0.803–0.856	5.4–6.3	0.829–0.849	5.5–5.8
*Medial deltoid*	0.760 ± 0.116	0.782 ± 0.134	0.893	5.3	0.795–0.836	6.6–7.3	0.811–0.868	5.9–7.0
*Posterior deltoid*	0.616 ± 0.121	0.637 ± 0.109	0.931	4.8	0.826–0.901	5.8–7.7	0.832–0.892	6.0–7.5
*Pectoralis major*	0.801 ± 0.112	0.820 ± 0.115	0.878	4.9	0.811–0.843	5.5–6.1	0.798–0.857	5.3–6.4
*Upper trapezius*	0.617 ± 0.104	0.626 ± 0.09	0.921	2.7	0.839–0.904	4.9–6.3	0.832–0.890	5.2–6.5
*Triceps brachii*	0.493 ± 0.118	0.529 ± 0.112	0.913	6.6	0.849–0.893	7.4–8.7	0.856–0.903	7.0–8.5

ICC, intraclass correlation coefficient; SEM%, standard error of measurement as percentage; $\bar{m}$, mean; SD, standard deviation.
